# Improving the therapeutic efficacy of gene therapy for duchenne muscular dystrophy (DMD) by evaluating and managing inflammation

**DOI:** 10.3389/fgene.2025.1569289

**Published:** 2025-06-09

**Authors:** Kadalraja Raghavan, Nobunao Ikewaki, Senthilkumar Preethy, Samuel J. K. Abraham

**Affiliations:** ^1^ Department of Paediatric Neurology, Jesuit Antonyraj memorial Inter-disciplinary Centre for Advanced Recovery and Education (JAICARE), Madurai, India; ^2^ Department of Medical Life Science, Kyushu University of Medical Sciences, Japan; ^3^ Institute of Immunology, Junsei Educational Institute, Nobeoka, Miyazaki, Japan; ^4^ Fujio-Eiji Academic Terrain (FEAT), Nichi-In Centre for Regenerative Medicine (NCRM), Chennai, India; ^5^ Centre for Advancing Clinical Research (CACR), University of Yamanashi - School of Medicine, Chuo, Japan; ^6^ Antony- Xavier Interdisciplinary Scholastics (AXIS), GN Corporation Co. Ltd., Kofu, Japan; ^7^ Mary-Yoshio Translational Hexagon (MYTH), Nichi-In Centre for Regenerative Medicine (NCRM), Chennai, India; ^8^ R & D, Sophy Inc., Japan; ^9^ Levy-Jurgen Transdisciplinary Exploratory (LJTE), Global Niche Corp., Wilmington, DE, United States; ^10^ Haraguchi-Parikumar Advanced Remedies (HARP), SoulSynergy Ltd., Phoenix, Mauritius

**Keywords:** duchenne muscular dystrophy (DMD), gene therapy, inflammation, immune reaction, adjuvant

## Abstract

Duchenne muscular dystrophy (DMD): is a rare, life-limiting genetic disorder for which no curative treatment currently exists. While various gene therapy approaches, some approved and others still under clinical investigation have been explored, they have not consistently produced the desired outcome and, in some cases, have been associated with serious adverse effects, including mortality. A critical factor we wish to highlight is the hostile inflammatory environment inherent to skeletal muscles’ pathology in DMD, which may be further aggravated by gene therapy, either due to the viral vector used or the gene component itself. Therefore, a comparative and detailed evaluation of inflammatory biomarkers between control and treatment arms in such clinical trials is essential to determine whether therapeutic benefits are being compromised by inflammation. Based on the implications of such hostile environment on the therapeutic outcome, adding a safer and efficacious management strategy to mitigate the inflammation during gene therapies is considered indispensable. Therefore, we recommend further research on adjuvant anti-inflammatory approaches to ensure safety and improvement of the therapeutic outcome of gene therapies for DMD.

Duchenne muscular dystrophy (DMD) is a life-limiting X-linked recessive disorder and the most common genetic muscle disease, affecting approximately one in 3,500 live male births (Goyal et al., 2021). It is caused by mutations in the DMD gene, including exon deletions, nonsense mutations, intra-exonic insertions/deletions, exon duplications, splice site defects, and deep intronic mutations. These mutations result in the absence or malfunction of the full-length dystrophin protein (Moriyama and Yokota, 2024). Dystrophin is a large structural protein essential for stabilizing the sarcolemma. In its absence, muscle fibers become highly susceptible to damage during contraction, leading to repeated cycles of necrosis and regeneration. Over time, this ongoing damage results in the replacement of muscle tissue with fat and fibrotic tissue.

It is now well established that inflammation plays a key role in the pathogenesis of DMD, accelerating both disease progression and fibrosis (Kharraz et al., 2014). Several mechanisms contribute to this inflammatory environment. The compromised sarcolemma integrity allows extracellular calcium to enter and intracellular signaling molecules to be released. Elevated cytosolic calcium activates calpain, which further triggers the NF-κB inflammatory pathway by promoting the degradation of its inhibitor, IκBα. This cascade stimulates the release of pro-inflammatory cytokines, disrupting muscle repair and contributing to the abnormal persistence of inflammatory cells such as macrophages. Additionally, mechanical stretching of the sarcolemma during muscle contraction activates nicotinamide adenine dinucleotide phosphate (NADPH) oxidases (NOX), resulting in the production of superoxide anion radicals and an increase in reactive oxygen species (ROS) (Miyatake et al., 2016). NF-κB, a key transcription factor activated in dystrophic muscle possibly due to elevated calcium and/or ROS regulates a broad array of genes involved in inflammation, including cytokines and chemokines. Furthermore, calcium-induced plasma membrane disruption or muscle fiber necrosis leads to the release of danger-associated molecular patterns (DAMPs) from the cytoplasm, perpetuating chronic inflammation. Among these, adenosine triphosphate (ATP), released due to myofiber fragility, activates P2 receptors on the plasma membrane, further amplifying the inflammatory response (Miyatake et al., 2016). The damaged and regenerating muscle tissue, along with supporting cell types such as fibroblasts and satellite cells, produces various soluble factors that exacerbate inflammation and disease progression (Kharraz et al., 2014).

Current therapeutic strategies for DMD include corticosteroids, which remain the standard first-line treatment. Investigational and emerging definitive therapies involve gene replacement approaches using viral vectors, plasmid-mediated nonviral delivery, stem cell transplantation, antisense oligonucleotide-induced exon skipping, and nonsense mutation suppression using pharmacological agents (Moriyama and Yokota, 2024; Konieczny et al., 2013). Among these, gene therapy has recently gained the most attention as the next-generation approach targeting the underlying pathology of DMD. The gene therapy includes mini-dystrophin and micro-dystrophin gene therapies, as well as CRISPR (Clustered Regularly Interspaced Short Palindromic Repeats) based techniques. In June 2023, Sarepta Therapeutics’ Elevidys (SRP-9001; delandistrogene moxeparvovec) received accelerated United States (US) Food and Drug Administration (FDA) approval as the first gene therapy/micro-dystrophin treatment for DMD in children aged 4–5 years (Konieczny et al., 2013). Elevidys employs an adeno-associated virus (AAV) vector of the rAAVrh74 serotype to deliver a truncated micro-dystrophin gene *via* a single intravenous infusion. Due to the limited gene-carrying capacity of AAV vectors, the full 2.4 Mbp dystrophin gene cannot be delivered; hence, a truncated mini- or micro-dystrophin protein is used to retain essential functionality. Elevidys gained accelerated approval based on increased micro-dystrophin expression in skeletal muscle and, in June 2024, received full approval for ambulatory children over 4 years of age and expanded accelerated approval for non-ambulatory children of the same age group. Ongoing clinical trials are further exploring mini-dystrophin/micro-dystrophin therapies from companies like Solid Biosciences, Regenexbio, and Genethon as well (Konieczny et al., 2013). In June 2024, Pfizer reported that its gene therapy drug, fordadistrogene movaparvovec (PF-06939,926), did not meet the primary endpoint in the phase III CIFFREO trial (NCT04281485) and the same product had raised safety concerns as well (Pfizer provides update on phase, 2005) and a subsequent discontinuation of the drug ensued. Although 30% of adverse events were considered manageable in a phase Ib trial (NCT03362502), leading to ‘Fast Track’ designation by the US-FDA, subsequent reports indicated that three patients experienced serious adverse events, including two cases of myocarditis. Additionally, one patient who received the drug passed away in December 2021 (Lek et al., 2023a). Reports of fatalities have also emerged in association with CRISPR-based gene therapies. In one case from September 2023, a 27-year-old DMD patient, diagnosed at age five and non-ambulatory by 18 with declining cardiopulmonary function, received rAAV9-dSaCas9-VP64 as an N-of-1 treatment. Six days post-treatment, the patient died from acute respiratory distress syndrome (Lek et al., 2023b). It is believed that the high-dose AAV vector induced a severe innate immune response, which likely contributed to the fatal outcome (Duan, 2023). The first approved Sarepta Therapeutics’ Elevidys (SRP-9001; delandistrogene moxeparvovec) in the recent report of EMBARK, a large, phase 3, two-part, multinational, randomized, double-blind, placebo-controlled trial also did not meet the primary endpoint (Mendell et al., 2024).

While these endpoints in most of the above clinical trials typically assess dystrophin expression and improvements in muscle function through measures such as the North Star Ambulatory Assessment (NSAA) score, Time to Rise, 10-m Walk/Run, stride velocity at the 95th percentile, and 100-m Walk/Run, *etc.*, we hypothesize that in the inflammatory and immune-reactive environment inherent to DMD, treatments may not achieve optimal efficacy unless this inflammation is effectively managed. The other aspect is the immunogenicity and inflammation induced by these gene therapies themselves either because of the viral vector alone, i. e., AAV or the gene component itself (dystrophin expression induced immunity) (Bönnemann et al., 2023; Schwotzer et al., 2024) or both, making the inflammation manifold (Kropf et al., 2024). This intensified inflammation may hinder the muscle regeneration process, which is crucial for the survival and differentiation of satellite stem cells carrying the newly introduced gene that differentiates into myofibers (Tripodi et al., 2021).

The immune response in gene therapy is driven by innate immunity and involves two primary mechanisms. Firstly, specialized receptors known as pattern recognition receptors (PRRs) such as Toll-like receptors detect conserved molecular patterns termed as pathogen-associated molecular patterns (PAMPs) on the invading viral vectors. This detection triggers the activation of key transcription factors, including NF-κB and interferon regulatory factors IRF3 and IRF7, which together drive the expression of proinflammatory cytokines and type I interferons in the infected cells, contributing to the host immune response (Schulz et al., 2023). Secondly, preformed or newly generated antibodies targeting viral vector antigens bind to these antigens, activate the classical complement pathway, and possibly the lectin pathway. Consequently, patients previously immunized against the viral vectors used for gene therapy are often ineligible, as viral vector administration could trigger a severe inflammatory response, while neutralizing antibodies reduce the efficiency of target cell transduction (Wang and Shao, 2023). Further, complement activation remains central to the toxicity mediated by neutralizing antiviral antibodies. The activation fragment iC3b, in particular, enhances viral vector opsonization and facilitates their uptake by monocytes and macrophages, triggering NF-κB-dependent inflammatory gene expression, such as IL-1β, IL-8, and macrophage inflammatory protein 2. The adaptive immune response then ensues, characterized by: (i) the activation of specific T and B cells reactive to viral antigens (in naïve patients) or to transduced gene products, and (ii) the subsequent release of inflammatory cytokines (Wang and Shao, 2023). In a recent gene therapy clinical trial report, it has been stated that the inflammation induced by pre-existing anti-AAV antibodies poses a risk for activation of the complement system (Potter et al., 2024), which may have led to heightened inflammation and also poses a significant safety concern (Mendell et al., 2024; Potter et al., 2024).

We therefore offer constructive suggestions that may help improve therapeutic efficacy of gene therapies by addressing the hostile inflammatory and immune-reactive environment within muscle tissue, particularly through the potential use of combination therapies that include strategies targeting inflammation.

Pharmacological corticosteroid therapy remains the standard of care for DMD, which primarily slows disease progression by reducing inflammation-mediated muscle damage (Kourakis et al., 2021). Currently used glucocorticoids, such as prednisone, prednisolone, and deflazacort, are effective but act in a non-selective manner which contributes to a range of side effects such as weight gain, growth retardation, behavioral problems, osteoporotic fractures, cataracts, delayed puberty, cushingoid appearance, etc., that can negatively affect the patient’s quality of life (Fontaine et al., 2024).

Next to corticosteroids, the use of commonly prescribed nonsteroidal anti-inflammatory drugs (NSAIDs), which broadly inhibit cyclooxygenase (COX) enzymes, have been studied but they have shown only limited therapeutic benefit in animal models. In preclinical studies, daily administration of NSAIDs such as aspirin and ibuprofen improved muscle morphology and reduced macrophage infiltration and necrosis. However, these treatments did not significantly alter the proportion of regenerating myofibers (Serra et al., 2012). Further, evidence from multiple placebo-controlled trials and meta-analyses has raised significant concern regarding the adverse effects of NSAIDs, which have been associated with complications involving the gastrointestinal, cardiovascular, hepatic, renal, cerebral, and pulmonary systems (Bindu et al., 2020).

To mitigate calcium overload–induced inflammation, therapeutic strategies targeting transient receptor potential (TRP) channels, key mediators of extracellular calcium influx due to their expression on the plasma membrane have been experimentally tried. Among these, Tranilast, a clinically approved antiallergic agent known to inhibit TRPV2 channels, has been studied in mdx mice, in which Tranilast administration led to a reduction in serum creatine kinase levels and skeletal muscle fibrosis, resulting in enhanced resistance to muscle fatigue (Iwata et al., 2005). Besides calcium influx, increased ROS production in DMD also represents a potential therapeutic target for anti-inflammatory intervention. One such approach involves inhibiting NOX, enzymes responsible for superoxide generation. Diapocynin, a dimer of the widely used NOX inhibitor apocynin, has been shown to prevent strength loss in skeletal muscles following eccentric contractions in mdx mice. At the level downstream of ROS generation, stabilizing ryanodine receptors (RyRs) in their closed state using Rycals RyR channel modulators has been effective in restoring both cardiac and skeletal muscle function by correcting RyR oxidation and overactivation (Andersson et al., 2012). However, potential adverse effects and long-term effects in humans require further investigation. Idebenone, an antioxidant compound, is currently undergoing clinical trials to evaluate its effectiveness in improving pulmonary function, motor performance, muscle strength, and overall quality of life in DMD patients who are not receiving corticosteroid therapy. Insomnia and mild liver transaminase abnormalities has been reported in other studies with Idebenone (Wang et al., 2024). NF-κB, a key transcription factor involved in regulating inflammatory pathways, represents a significant target for anti-inflammatory therapy in DMD. CAT-1004, an NF-κB inhibitor, has successfully completed Phase I clinical trials. However, NF-κB inhibitors can have several adverse effects, including immunodeficiencies, hepatotoxicity, and an increased risk of certain cancers, especially in the liver (Betzler et al., 2020). Vamorolone (VBP15), a dissociative corticosteroid, a compound structurally related to glucocorticoids that retains anti-inflammatory efficacy without the typical steroid-associated side effects has shown therapeutic potential in mdx mouse models (Heier et al., 2013) and has also demonstrated clinical efficacy in DMD over 48 weeks (Dang et al., 2024). It has also received USFDA approval for use in DMD patients aged 2 years and older (ajmc, 2025). *In vivo* inhibition of the extracellular ATP/P2X purinergic signaling pathway using periodate-oxidized ATP has been shown to delay disease progression and alleviate local inflammation in mdx mice. This intervention resulted in reduced leukocyte infiltration and decreased interleukin-6 (IL-6) expression, indicating a dampened inflammatory response (Gazzerro et al., 2015). Elevated levels of IL-6 have been detected in both the circulation and muscle tissue of DMD patients and young mdx mice, highlighting its role in disease-associated inflammation. Over the past decade, the US-FDA has approved several novel agents targeting IL-6 signaling for the treatment of various inflammatory conditions. IL-6 inhibitors can cause side effects like infections, neutropenia, and abnormalities in liver enzymes (Ogata et al., 2019). Given their established anti-inflammatory effects, these drugs hold potential for therapeutic application in DMD (Miyatake et al., 2016). In contrast to strategies aimed at suppressing inflammation, another therapeutic approach involves enhancing the anti-inflammatory response and promoting muscle growth through insulin-like growth factor-1 (IGF-1) signaling in skeletal muscles. A Phase II clinical trial of recombinant IGF-1 (INCRELEX™) has been launched in glucocorticoid-treated DMD patients to assess its potential in preserving muscle function over a 6-month period. Fluid retention, gynecomastia, and orthostatic hypotension are associated side effects (Sullivan et al., 1998).

Immunosuppressive multipotent mesenchymal stromal cell (MSC) therapy is emerging as a potential therapeutic approach for mitigating inflammation in patients with DMD. Preclinical studies have shown promising outcomes; for instance, early systemic administration of human amniotic MSCs (hAMSCs) in mdx mice ameliorated progressive DMD phenotypes, reduced pathological inflammation, improved motor function, and led to sustained improvement in both skeletal and cardiac muscle performance (Nitahara-Kasahara et al., 2023). A recent open-label, dose-escalation Phase 1 clinical trial evaluated the safety and dosage of EN001, an allogeneic, early-passage Wharton’s jelly-derived MSC product developed by ENCell, in DMD patients (Lee et al., 2025). The trial results indicated that EN001 was safe and well tolerated, with no serious adverse events reported. While these findings support the feasibility of MSC-based therapy for DMD, the clinical efficacy of EN001 remains to be fully established in larger trials. Despite encouraging results, several limitations exist in the current MSC-based therapeutic approaches. One major challenge is the short-term survival of transplanted naïve MSCs *in vivo*, inability to detect transplanted MSCs in host tissues after administration, suggesting limited persistence and the fact that most studies have focused on local injections, which may not be adequate given the systemic nature of DMD (Shen et al., 2021). Figure 1 summarizes the pros and cons of different anti-inflammatory strategies in DMD.

**FIGURE 1:**
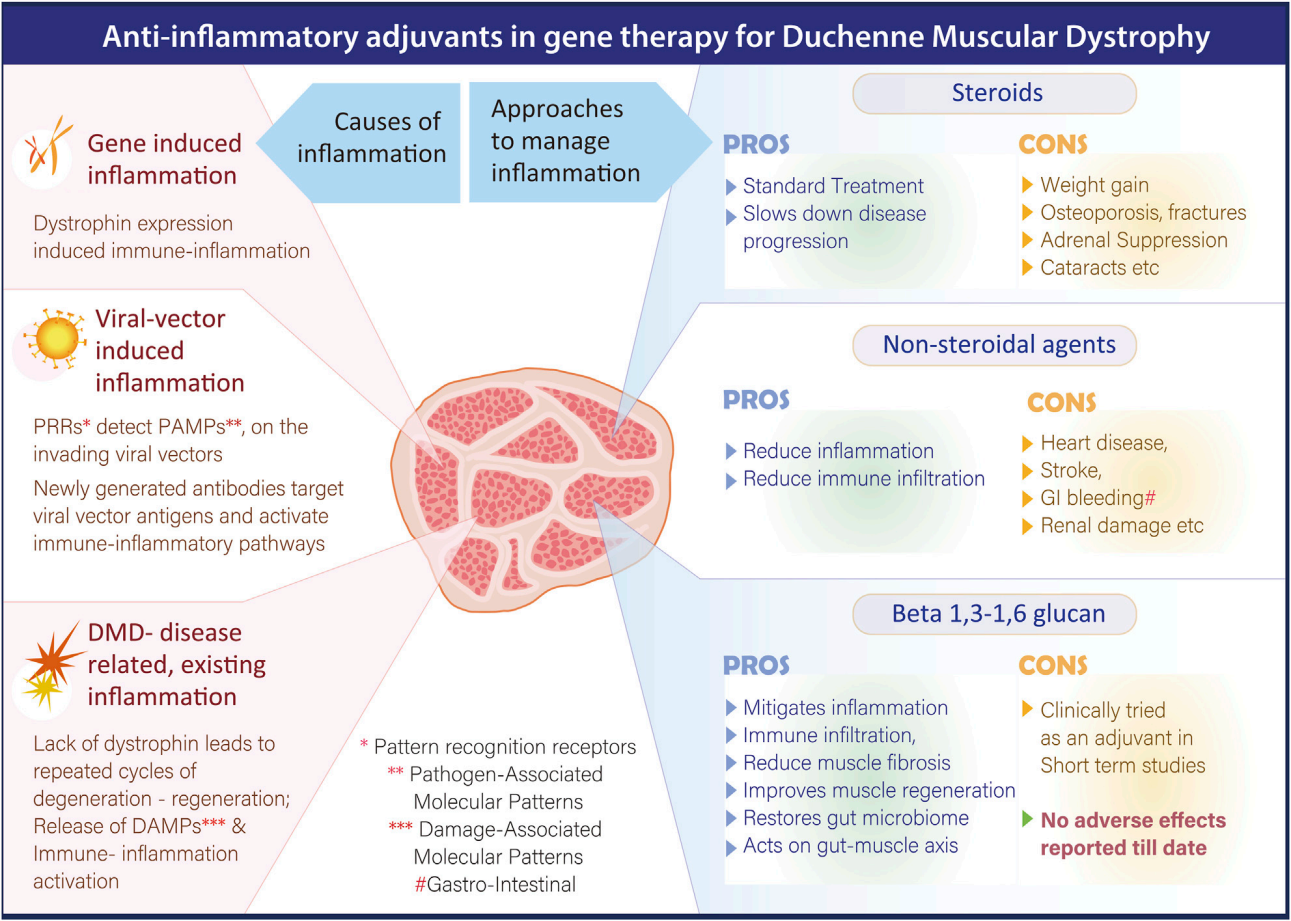
Overview summarizing the pros and cons of different anti-inflammatory strategies in DMD.

It is also important to note that such anti-inflammatory interventions, while mitigating the inflammation, may further impair the immune response (Cordova et al., 2018), which may lead to aggravation of viral-induced inflammation or gene-related immunogenicity. Also, these approaches have not yet been studied in combination with gene therapy.

Therefore, in light of the associated adverse effects apart from impaired immune response with the above strategies targeting inflammation, we would like to bring forth the pre-clinical and clinical outcome of a safe, orally administrable biological response modifier adjuvant, a black yeast Aureobasidium pullulans N-163 strain produced 1,3-1,6 beta-glucan beta-glucan in studies for DMD. This adjuvant has effectively reduced plasma biomarkers of inflammation in human clinical studies [IL-6, IL-13, serum calcium, creatine phosphokinase (CPK) and alkaline phosphatase (ALP)] (Raghavan et al., 2023), alanine transaminase (ALT), aspartate transaminase (AST), lactate dehydrogenase (LDH), IL-13 and fibrosis in mdx mice (Preethy et al., 2023a). Further, in mdx mice, this beta-glucan has also shown to increase skeletal muscle regeneration markers: plasma IGF-1, Dystrophin, MYH3 and CD44 apart from increasing CD68 macrophages infiltration that have the ability to clear necrotic muscle (Preethy et al., 2025). Decrease in inflammation associated CD93+ve cell infiltration in diaphragm and myocardial fibrosis have also been reported (Preethy et al., 2023b; Abraham et al., 2025). Immune-inflammatory modulation through beneficial reconstitution of the gut microbiome in DMD by this beta-glucan has also been reported (Raghavan et al., 2022). With hepatic toxicity also been reported in clinical trials of gene therapy (Duan, 2023), this adjuvant approach may also help in mitigating this hepatic inflammation and fibrosis, as it has been reported earlier with relevant beneficial implications in non-alcoholic steatohepatitis (NASH) animal models (Ikewaki et al., 2022). Figure 2 illustrates the pathways through which beta 1.3-,1,6-glucan adjuvant mediates its beneficial anti-inflammatory effects across the various stages of DMD progression.

**FIGURE 2:**
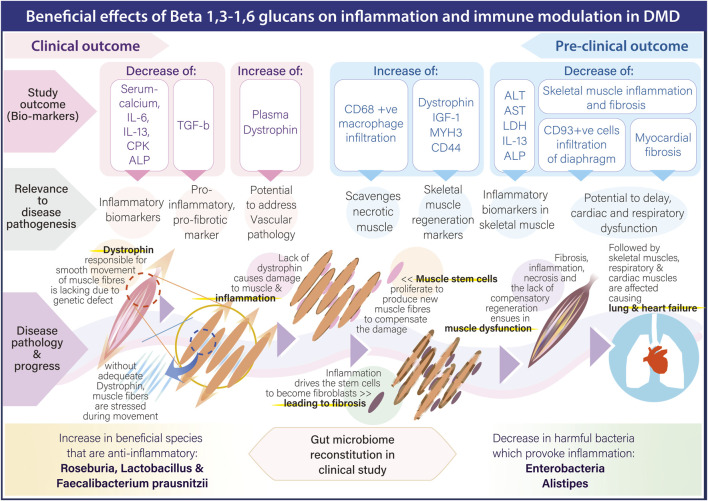
Pathways through which beta 1.3-,1,6-glucan adjuvant mediates its beneficial anti-inflammatory effects across the various stages of DMD progression.

We suggest comparative animal studies be undertaken using such anti-inflammatory approaches of different combinations along with gene therapies and it is worthwhile to mention that the safety of adjuvant therapies along with steroids of this N-163 beta-glucan has been studied in a clinical trial earlier, though in small numbers for a short duration (Raghavan et al., 2023). Such evaluation of safety and efficacy of each such approach in enhancing the therapeutic outcome in AAV based gene therapy clinical trials is recommended as a way forward.

The definitive remedial approach of gene therapies for DMD have generally overlooked the assessment of both inherent inflammation and treatment-induced immune responses between control and treatment arms in the clinical trials reported till date. Having known that the hostile inflammatory immune-reactive environment is detrimental to a constructive regeneration of the muscle cells post gene therapy, along with the evaluation of such hostile environment during such clinical trials, ways to manage them are also indispensable to a bring a beneficial or clinically efficacious outcome. While anti-inflammatory adjuvants along with gene therapy may have their own shortfalls, at least evaluation of the inflammatory biomarkers would give some insight into whether such inflammation has primarily negatively impacted the outcome. While steroids help reduce overall inflammation and other targeted approaches provide anti-inflammatory effects, each of these strategies have their own limitations in terms of safety and efficacy. We therefore recommend evaluating varied combinations of these anti-inflammatory approaches, including the novel beta 1.3–1.6 glucans as adjuvants to gene therapies in long-term studies to find which of them would safely and effectively improve the therapeutic outcome.
